# Rapid and Label-Free Immunosensing of Shiga Toxin Subtypes with Surface Plasmon Resonance Imaging

**DOI:** 10.3390/toxins12050280

**Published:** 2020-04-26

**Authors:** Bin Wang, Bosoon Park, Jing Chen, Xiaohua He

**Affiliations:** 1 USDA, ARS, SEA, USNPRC, 950 College Station Rd, Athens, GA 30605, USA; 2 Food Science Center, Merieux NutriSciences (China), Shanghai 201112, China; 3 USDA, ARS, PWA, WRRC, 800 Buchanan Street, Albany, CA 94710, USA

**Keywords:** Surface plasmon resonance imaging, Shiga toxin, foodborne pathogen, label-free detection, nanoparticle, sandwich immunoassay, food safety

## Abstract

Shiga toxin-producing *Escherichia coli* (STEC) are responsible for gastrointestinal diseases reported in numerous outbreaks around the world as well as in the United States. Current detection methods have limitation to implement for rapid field-deployable detection with high volume of samples that are needed for regulatory purposes. Surface plasmon resonance imaging (SPRi) has proved to achieve rapid and label-free screening of multiple pathogens simultaneously, so it was evaluated in this work for the detection of Shiga toxins (Stx1a and Stx2a toxoids were used as the less toxic alternatives to Stx1 and Stx2, respectively). Multiple antibodies (Stx1pAb, Stx1-1mAb, Stx1-2mAb, Stx1d-3mAb, Stx1e-4mAb, Stx2pAb, Stx2-1mAb, Stx2-2mAb, and Stx2-10mAb) were spotted one by one by programed microarrayer, on the same high-throughput biochip with 50-nm gold film through multiple crosslinking and blocking steps to improve the orientation of antibodies on the biochip surface. Shiga toxins were detected based on the SPRi signal difference (ΔR) between immobilized testing antibodies and immunoglobulin G (IgG) control. Among the antibodies tested, Stx1pAb showed the highest sensitivity for Stx1 toxoid, with the limit of detection (LOD) of 50 ng/mL and detection time of 20 min. Both Stx2-1mAb and Stx2-2mAb exhibited high sensitivity for Stx2 toxoid. Furthermore, gold nanoparticles (GNPs) were used to amplify the SPRi signals of monoclonal antibodies in a sandwich platform. The LOD reached the level of picogram (pg)/mL with the help of GNP-antibody conjugate. This result proved that SPRi biochip with selected antibodies has the potential for rapid, high-throughput and multiplex detection of Shiga toxins.

## 1. Introduction

Shiga toxin-producing *Escherichia coli* (STEC) are responsible for gastrointestinal diseases reported in numerous outbreaks around the world. The Centers for Disease Control and Prevention (CDC) estimates that each year STEC causes 265,000 illness, 3600 hospitalizations, and 30 deaths in the United States alone [1]. Among 5–10% of these patients, the *E. coli* O157 infection will develop a potential neurological involvement in hemolytic uremic syndrome (HUS), a type of kidney failure [2]. Current detection methods include culture enrichment, real-time PCR, and enzyme immunoassay (EIA) [2]. Although each method has advantages over others in terms of specificity and sensitivity, it is difficult to use one technique platform for rapid detection of STEC or Shiga toxins (Stx) contaminating in samples directly obtained from the food industry and market, where the rapid screening and identification of foodborne pathogens are frequently demanded by regulatory agencies. Our previous study demonstrated an optical method with surface plasmon resonance imaging (SPRi) that has the potential for rapid and label-free screening of multiple pathogenic bacteria simultaneously [3]. Here we expanded the label-free SPRi detection to the immunosensing of Shiga toxins (Stx1, Stx2) produced by STEC. Compared to conventional cell-culture based methods, SPRi has the benefit of detecting targets faster, field-portably, and highly multiplexably. While compared to conventional immunoassays, such as enzyme-linked immunosorbent assay (ELISA), SPRi is easier to operate, label-free, portable, and has higher throughput. Among different types of Stxs discovered, Stx1a and Stx2a are the most common ones associated with human diseases [4]. According to methods approved by FDA, real-time PCR can detect the genes of Stx1, Stx2, and uidA single-nucleotide polymorphism in *E. coli* O157:H7 strain down to 6 CFU/reaction within 40 PCR cycles [2,5]. Although real-time PCR showed very high sensitivity, the PCR method is labor intensive and requires highly skilled professionals to operate, with the total sample preparation and detection time in the range from hours to days. Therefore, this study exploited a label-free and high-throughput SPRi platform to develop the immunosensor with rapid detection time of less than 20 min while maintaining high specificity and sensitivity.

Our previous work showed that mAb Stx1-2 is a good capture antibody and mAb Stx1-1 is a good detection antibody for Stx1a toxoid (simplified as Stx1a*) [6]; while, for Stx2a toxoid (simplified as Stx2a*), mAb Stx2-1 is good as a capture antibody and mAb Stx2-2 is good as a detection antibody [7]. In addition, Stx2a, Stx2c, and Stx2d have also been frequently linked to the development of HUS, and Stx2e has been proven to cause edema disease in pigs and mild diarrhea in human HUS patients [8]. Therefore, the ability to identify all subtypes of Stxs is critical in surveillance programs. In order to detect all 10 subtypes of Shiga toxins produced by STEC in ground beef, a universal sandwich ELISA has been developed and tested to detect Stx1 and Stx2 [9]. In that study, a mAb cocktail containing antibodies Stx1-2, Stx2-5, Stx2b-1, Stx2e-2, and Stx2f-1 was used to capture the Stx, while a mixture of anti-Stx1 and anti-Stx2 polyclonal antibodies was used for detection with additional horseradish peroxidase conjugated goat anti-rabbit immunoglobulin G (IgG-HRP) as the secondary antibody. The limit of detection (LOD) for different subtypes was between 10 and 50 picogram (pg)/mL. Currently, two commercial Stx1 and Stx2 ELISA kits (Abraxis Inc., Warminster, PA, USA) are available to detect all 10 subtypes of Stx1 and Stx2, with the LOD of 25 pg/mL. However, these ELISAs require bulky instruments and extensive sample preparation processes, which hinder ELISA applications in the field. On the contrary, SPRi microarrays operate in a flow channel system that can be integrated with versatile sample injection and detection devices. Therefore, SPRi is the high-throughput and multiplex platform that can be utilized in both the research lab and real world, such as the field-deployable detection/sensing in the food industry or agriculture.

In this study, we developed a label-free and easy-to-use SPRi immunosensor capable of high-throughput microarray detection of Stx1 and Stx2. To reduce biohazardous materials, less toxic alternatives of Stxs, the recombinant toxoids Stx1a* and Stx2a*, were used as targets during assay development. The antibodies against Stx1 and Stx2 were selected according to their performance in SPRi screening to form the sandwich immunoassay for identification of targets on SPRi high-throughput biochips. In the future, based on this study, more tests will show the detection of Stx in spiked serum samples and in fecal/food extracts, which will focus on the further improvement on sensitivity and robustness. Depending on the future development, this SPRi sandwich immunoassay could become a powerful tool for screening of agricultural products contaminated with foodborne pathogens and toxins.

## 2. Results and Discussion

### 2.1. Detection of Stx with Direct Label-Free Immunoassay

**Figure 1:**
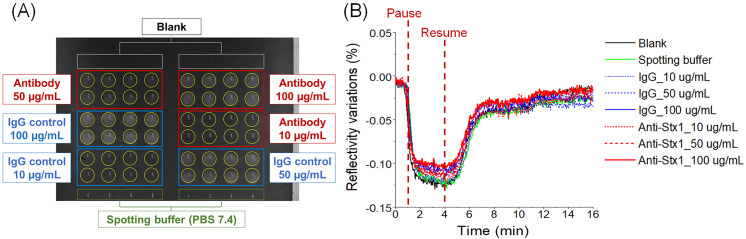
Label-free detection of Stx1a* at 100 ng/mL. (**A**) Real-time biochip image of each antibody spot, which is highlighted by circle in yellow. (**B**) SPRi sensorgram of Stx1a* to different antibodies and controls. The 3-min pause-resume injection provides 3 min of additional reaction time for antibodies to distinguish the toxoid.

The entire biochip was covered with mercaptoundecanoic acid (MUA), and subsequently activated by 1-ethyl-3-(3-dimethylaminopropyl) carbodiimide (EDC) and N-hydroxysuccimide (NHS) coupling reagents (EDC-NHS), and spotted with antibodies by the microarrayer capillary pin with the diameter of 500 µm. The real-time SPRi images of spotted microarray are shown in Figure 1A. The SPRi sensorgrams, including each spotted group labeled in Figure 1A, are shown in Figure 1B with injection of 100 ng/mL Stx1a*. For Stx1a* and Stx2a*, sample concentrations lower than 100 ng/mL did not show significant difference from IgG control (data not shown).

**Figure 2:**
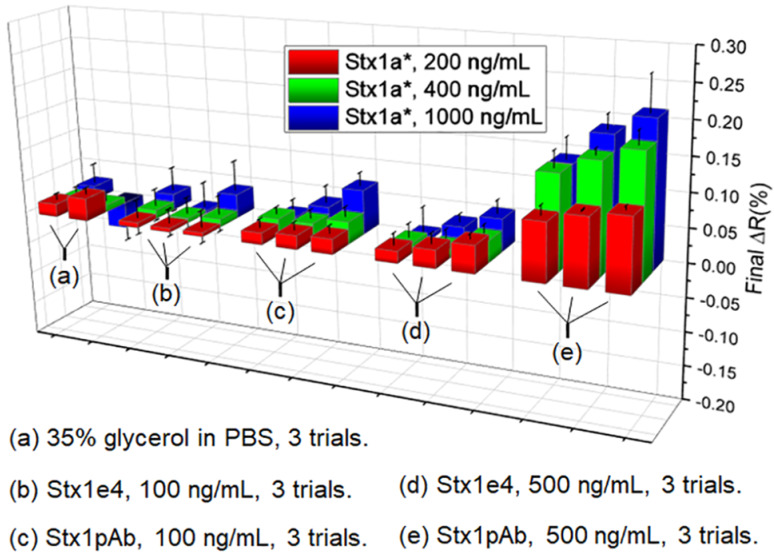
Summary of label-free detections of Stx1a* with various antibodies from (**a**) to (**e**) with strongest signal from the Stx1pAb, and weakest signal from the mAb Stx1e-4. Stx1a* represents the Stx1a toxoid.

Various antibodies against Stx1a and Stx2a have been tested in SPRi. For Stx1a*, the polyclonal antibody Stx1pAb provided the strongest SPRi difference signals (ΔR, with artificial unit %) over IgG control when the toxoid concentration was in the range of 200 ng/mL to 1.0 mg/mL (Figure 2). Therefore, Stx1pAb could be a good candidate for future SPRi biochip to detect Stx1 and Stx1-producing STEC in food samples.

**Figure 3:**
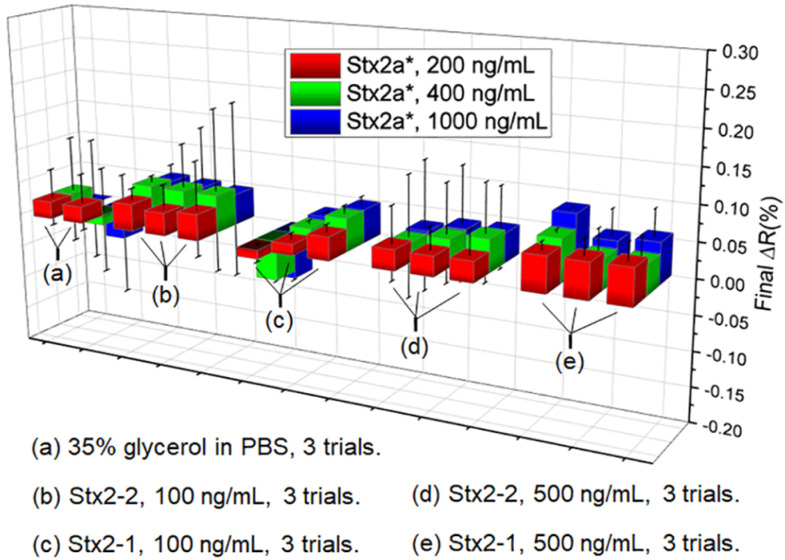
Summary of the label-free detection for Stx2a* with various antibodies (**a**–**e**). Both mAb Stx2-1 and Stx2-2 detected Stx2a* with relatively weak signals. Stx2a* represents the Stx2a toxoid.

The monoclonal antibodies against Stx1a* (in Figure 2) and against Stx2a* (in Figure 3) have shown weak ΔR signals against IgG control with large standard deviation (SD) values. The LOD was 100 ng/mL, which is larger than the LOD of ELISA using the same antibodies [6,7]. One explanation is that the label-free signals from SPRi fully depend on the mass and refractive index of target molecules. The Stx proteins have relatively small size (~70 kDa), so the SPRi response from Stx toxoid binding is not as strong as cells (with large mass) or metal particles (with significant optical properties). Therefore, additional signal amplification may be required before the SPRi method can be used for detection of Stxs in real-world samples. The detail of this work is shown in Section 2.2.

**Figure 4:**
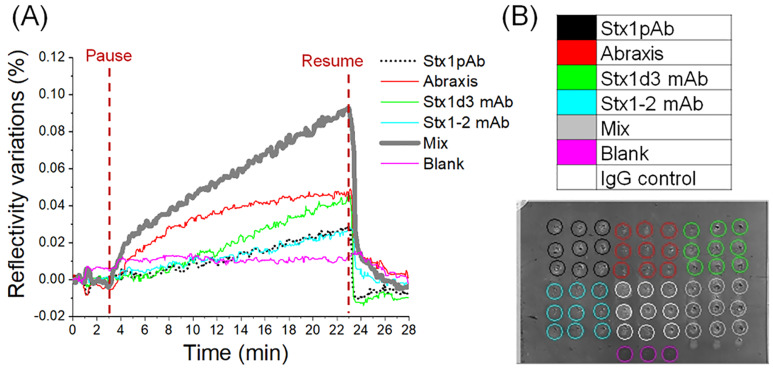
Label-free detection of Stx1a* at 50 ng/mL. (**A**) The SPRi sensorgram of Stx1a* to different antibodies. Each sensorgram curve is the difference signal over IgG control. (**B**) The real-time biochip image with the labeled spots corresponding to the sensorgrams in (**A**). The signal from IgG (in white circles) is already subtracted from each curve in (**A**). In both (**A**) and (**B**), Mix is the mixture of Stx1pAb, Stx1-2 mAb, and Stx1d-3 mAb.

Another test using a mixture of three antibodies (Stx1d-3, Stx1-2, and Stx1pAb) spotted on biochip surface under the same condition showed that the antibody mixture detected Stx1a* down to 50 ng/mL (Figure 4), a four-fold increase in sensitivity compare with the assay using Stx1pAb alone. Here the pause-resume injection was 20 min to increase the antibody-binding probability. However, the mixture of four Stx2 antibodies (Stx2-1, Stx2-2, Stx2-10, and Stx2 pAb) did not show repeatable specific signals (data not shown). Therefore, in this research stage, the mixture of three antibodies provided the most sensitive detection for SPRi label-free detection, although it was only valid for Stx1a*. In CDC-approved diagnosis method [10], the concentration of free fecal Stx in patients was very low (at pg/mL level) and did not correlate with the Stx expressed by bacteria grown in vitro and was not related to bacterial titer in the studied samples [11]. Although the assay in this study was less sensitive compared with our previous ELISA [9], the SPRi approach was still useful for field applications due to its fast (<20 min) and label-free features.

### 2.2. Signal Amplification with Gold Nanoparticle (GNP)

The GNPs have been successfully used in plasmon-based biosensors to amplify the signals. The major advantages of GNP over other reagents are its high sensitivity, relatively easy and inexpensive synthesis, unique optoelectronic properties, and high surface-to-volume ratio [12,13,14,15]. Therefore, GNP has been commercialized for versatile applications in bioimaging and biosensing [16,17]. Based on previously developed methods in literature, GNP was chosen to enhance the SPRi signals in a sandwich format in order to detect low concentrations of Stx present in real-world samples.

The GNP used here was functionalized GNP. The surface of GNP was coated with polyethylene glycol with average molecular weight of 2000 Da (PEG2000) linker molecules to provide reaction freedom of the secondary antibody, which was attached to the top of the PEG linker. This functionalized GNP reagent was ready for one-step reaction of the secondary antibody binding to the fragment crystallizable (Fc) of the anti-Stx antibodies, so that the antigen binding fragment (Fab) of the antibody could properly reach to the solution and maintain its activity. This orientation control of the antibody to the Stx molecules in solution is shown in Figure S1. The antibodies on the biochip surface were spotted by the capillary pin with the diameter of 700 µm. Our tests showed that the changing of pin diameter from 500 µm to 700 µm could improve the repeatability of the size and shape of each antibody spot on the gold film.

**Figure 5:**
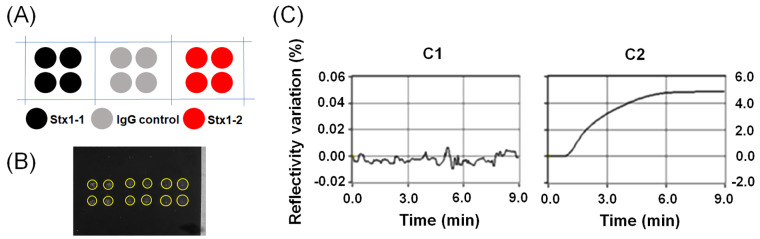
SPRi for sandwich immunoassay with (**A**) microarray pattern, (**B**) real-time SPRi image of the design pattern in (**A**), with each printed antibody spot highlighted by circle in yellow, and (**C**) the comparison of GNP-based SPRi signal amplification generated by the spots in (**B**), with (**C1**) showing the signal after Stx1a* injection but before GNP-Stx1-1 conjugate injection, and (**C2**) the signal after injection of GNP-Stx1-1 conjugate. More details are described in Section 4.4. Surface Plasmon Resonance Imaging.

The microarray pattern spotted using 700-µm pin is shown in Figure 5A, and its real-time SPRi image is shown in Figure 5B. The larger spot size printed with 700-µm pin limited the number of spots the biochip surface can contain, but it was still a high-throughput microarray capable of multiplex detections. The comparison in Figure 5C shows that the signal of Stx1a* was almost buried in baseline without GNP-Stx1-1 conjugate, but quickly increased two orders of magnitude within 6 min after the injection of GNP-Stx1-1 conjugate, without the use of pause-resume injection method. The total detection time from toxoid injection to signal generation was as short as 16 min, which can be further shortened by optimizing the operation protocol. This easy-to-use feature of SPRi approach is critical for the field-deployable detections.

**Figure 6:**
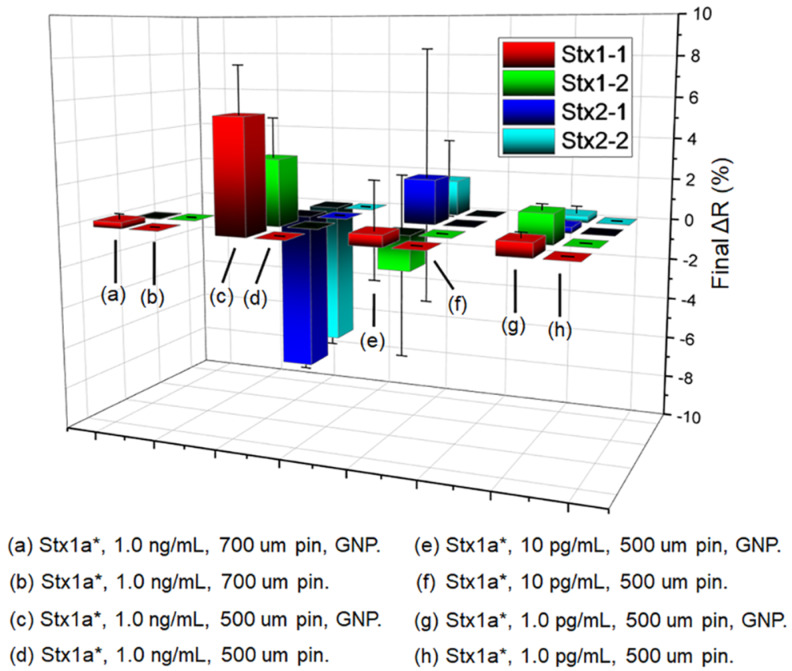
Summary of the signal amplification of Stx1a* at 1.0 pg/mL, 10 pg/mL, and 1.0 ng/mL by the sandwich immunoassay consisting the GNP-Stx1-1 conjugate and different antibodies (**a**–**h**), spotted by either 500-µm pins or 700-µm pins on biochip. The Final ΔR is the reflection difference between each antibody spotted and the IgG spotted as the control. Stx1a* represents the Stx1a toxoid.

The signal amplifications on different antibody spots are summarized in Figure 6. Here the GNP-Stx1-1 conjugate solution injected after toxoid was optimized as 1 nM, because GNP at too high concentration caused very large background signal, while GNP at too low concentration did not provide significant signal amplification. Even though, the background signals from GNP on other surface areas of the biochip always interfered with the specific signals from the sandwich immunoassay, and in turn generated large standard deviation values for those antibody spots on the biochip. Especially, IgG as control could interact with GNP or secondary antibody to generate nonspecific signals when the sandwich immunoassay was formed on the biochip surface. The “Final ΔR” values calculated from the difference between Stx antibodies and IgG control in Figure 6 were essentially determined by how large the control signal was. Therefore, in Figure 6, the signal from (g) “Stx1a*, 1.0 pg/mL, 500 µm pin, GNP.” is higher than the one from (e) “Stx1a*, 10 pg/mL, 500 µm pin, GNP.”. This large deviation was not from the specific signals of the sandwich immunoassays, but from the IgG control signals of those two injections. The high signals from IgG control also caused large negative values of the blue and cyan columns in Figure 5 sample injection (c). This problem implies that the GNP sandwich assay still had issues of unstable structure and nonspecific binding. The standard deviation values on some columns in Figure 6 are very large, which were caused by the same issue. The SPRi sandwich immunoassay had a relatively complex mechanism depending on the interactions among multiple antibodies and nanoparticle, so the system errors in the final signals were generally larger than the simple SPRi label-free detections. This difference is reflected in the values of standard errors in Figure 6.

In Figure 6, the best amplification effect for Stx1a* was generated when the mAb Stx1-1 was used as the capture antibody (as the bottom layer) on the biochip surface and the GNP conjugated Stx1-1 was used as the detection antibody (as the top layer). This antibody combination for the sandwich assay is different from our previous ELISA, where the Stx1-2 antibody was used as capture antibody and Stx1-1 antibody was used as the detection antibody [7,9,18]. It indicated that the antibodies optimized in ELISA may not be the best choice in SPRi immunoassay, due to difference in assay platform and conditions. Our results demonstrated that GNP-based immunoassay was rapid and sensitive for detection of Stx1a*. The surface modifications and antibody-binding designs adopted from ELISA are helpful but need to be carefully tested in SPRi platform and modified, if necessary, for better performance.

## 3. Conclusions

The Stx alternatives, Stx1a* and Stx2a*, were used in this study to test the SPRi immunoassay for detection of Stx in a flow cell system. Different monoclonal and polyclonal antibodies were tested by rapid label-free SPRi screening within 20 min. The mixture of three antibodies on the printed microarray spots exhibited the highest sensitivity at the ng/mL level without any labeling or enriching treatment. Based on the results, a GNP-based sandwich immunoassay was designed using four monoclonal antibodies against Stx1a and Stx2a, which significantly amplified the SPRi signals and improved the assay sensitivity (from ng/mL to pg/mL). Thus, the SPRi immunosensing method has potential to be used in the multiplex high-throughput detection of different Stxs produced by pathogenic bacteria. This study validated the SPRi platform, the pairs of antibodies for the immunosensor, and signal amplification by GNPs. Additionally, the Stx1 antibodies showed better specificity than Stx2 antibodies, which indicates that Stx2 antibodies are more affected by the spotting process and binding conditions on biochip surface. When adopting immunoassay from ELISA to SPRi, careful scrutinization is necessary to test the performance of different antibodies on the same biochip. The future study will focus on improving sensitivity and selectivity of detecting Stx in complex food matrix with advanced methods and techniques, where the sensitivity to meet regulatory requirement is the biggest challenge.

## 4. Materials and Methods

### 4.1. Materials

SPRi Biochips^TM^ were purchased from Horiba Scientific (Edison, NJ, USA). The surface modification reagents MUA, EDC, and NHS were obtained from Sigma-Alrich (St. Louis, MO, USA). The linker molecule HS-PEG2000-COOH was purchased from Nanosoft Polymers (Winston-Salem, NC, USA). Protein A/G and bis-(sulfosuccinimidyl)-suberate (BS3) were purchased from Thermo Scientific (Waltham, MA, USA). The skim-milk powder, bovine serum albumin (BSA), and Trizma^®^ hydrochloride were obtained from Sigma-Alrich (St. Louis, MO, USA).

Regeneration reagent for antibody screening is NaOH, purchased from Spectrum Chemical Mfg. Corp. (New Brunswick, NJ, USA). The regeneration reagents for GNP sandwich immunoassay include three steps: (1) Surfactant Tween 20, purchased from Millipore Sigma (St. Louis, MO, USA), (2) alkali solution consisting of NaOH purchased from Spectrum Chemical Mfg. Corp. (New Brunswick, NJ, USA) and sodium dodecyl sulfate (SDS) purchased from Fisher Scientific (Hampton, NH, USA), and (3) acidic solution consisting of HCl purchased from Fisher Scientific (Hampton, NH, USA), glycine purchased from Sigma-Alrich (St. Louis, MO, USA),) and dimethylformamide (DMF) purchased from Thermo Fisher Scientific (Waltham, MA, USA).

Phosphate buffered saline (PBS) and mouse IgG control were purchased from Thermo Fisher Scientific (Waltham, MA, USA). The Stx1a* and Stx2a* were purchased from Abraxis Inc. (Warminster, PA, USA). Stx1a* and Stx2a* were produced as described in previous publications [7,9]. Specifically, two toxoids were recombinant proteins with point mutation at position 167, where the glutamic acid was altered to glutamine. Therefore, Stx1a* and Stx2a* maintained general structural integrity, and their activity to antibodies were as close as the Stx1a and Stx2a toxins, but much less toxic. Shiga toxin monoclonal antibodies Stx1-1mAb, Stx1-2mAb, Stx2-1mAb, Stx2-2mAb, Stx1d-3mAb, Stx1e-4mAb, and Stx2-10mAb were generated by the same methods as previously described [6,7,9]. The polyclonal antibodies Stx1pAb and Stx2pAb were purchased from Abraxis Inc. (Warminster, PA, USA). The GNP used in this study was 30 nm in diameter, of which the surfaces were immobilized with the secondary antibody molecules via a condensed PEG2000 monolayer, as shown in Figure S1. The secondary antibody was the Fc-binding anti-mouse antibody, which can specifically bind to the Fc fragment of the Stx antibodies, so that the Fab fragments were free to catch the Stx toxoid on biochip surface and form the sandwich immunoassay (Figure S1). This functionalized GNP and GNP without secondary antibody (GNP control) were purchased from Nanopartz Inc. (Loveland, CO, USA).

Mention of trade names or commercial products in this article is solely for the purpose of providing specific information and does not imply recommendation or endorsement by the U.S. Department of Agriculture. 

### 4.2. SPRi Biochip Fabrication

The direct immobilization of Stx antibodies on the biochip surface was performed following the protocol 1 (Figure S2A). The biochip was immersed into 2 mM MUA EtOH solution to obtain a self-assembled monolayer (SAM) overnight. Next morning, the biochip was extensively flushed with EtOH 3 times and triple deionized (DI) water 3 times, the modified biochip surface was then activated by the mixture solution of EDC and NHS, 150 mM in DI water, respectively. Within 30 min, the reaction was terminated by a quick flushing with DI water and different antibodies and control IgG solutions were spotted according to the biochip design [19]. The spotting antibody was in glycerol:water = 1:4 (*v*:*v*) solution, with concentrations ranging from 0.25 to 1.0 mg/mL. The primary amine groups from the antibody molecules were attached to the MUA linker molecules by the EDC-NHS coupling reaction. After incubation for 2 h inside the spotting chamber, the surface was flushed with DI water 3 times. Finally, the blocking solution, 0.1% (*w*:*v*) BSA in PBS pH 7.4, was dropped on the biochip surface and incubated for 2 h to block unreacted NHS ester groups on the chip. After extensively flushing with PBS and DI water, the biochip was dried with nitrogen before being mounted inside the SPRi instrument (Horiba Scientific, Edison, NJ, USA).

In order to improve the antibody-binding efficiency on the biochip surface, new conjugation methods were used as protocol 2 (Figure S2B) [20]. The details of the protocol for surface immobilization were as follows. (1) The functionalized PEG2000 linker molecules, 10 mg/mL in glycerol:water = 1:4 (*v*:*v*) spotting solution, were spotted on the gold surface according to the microarray pattern (shown in Figure 5). The biochip was incubated inside the spotting chamber (LabNEXT, West New York, NJ, USA) for 2 h and flushed with DI water 3 times. (2) The biochip was immersed into 2 mM MUA EtOH solution and treated with EDC-NHS solution, the same as protocol 1 described, aforementioned. (3) Protein A/G, 1 mg/mL in PBS pH 7.4, was spotted on the same locations of the PEG2000 microarray spots, so that the protein A/G could specifically bind to the Fc fragments of each antibody molecule. After 2 h of incubation inside the spotting chamber, the biochip surface was flushed with PBS 3 times. (4) Then, 0.1% (*w*:*v*) BSA in PBS was used to block other areas of the biochip surface for 2 h, and the surface was extensively flushed to remove noncovalent binding. (5) Antibodies and IgG control, 1 mg/mL in glycerol:water = 1:4 (*v*:*v*) spotting solution, were spotted to the biochip as described in protocol 1. (6) The BSA 1 mg/mL in PBS solution was dropped on the biochip surface to covalently link antibody molecules to the protein A/G molecules. The biochip was incubated for 2 h and then flushed with PBS 3 times before being mounted into SPRi instrument for sample measurement.

In each spotting step of protocol 1 and 2, the XactII™ compact microarray spotter (LabNEXT, West New York, NJ, USA) was placed inside a chamber with controlled humidity greater than 75%. For protocol 1, the capillary pin used for spotting had the spotting volume of 5 nL/spot with a diameter of 500 µm. For protocol 2, the pin had the spotting volume of 10 nL/spot with a diameter of 700 µm.

### 4.3. Synthesis of GNP-Antibody Conjugate

In order to amplify the specific signals from antibody-toxoid binding, a sandwich immunoassay was designed, in which the capture antibodies were spotted on the biochip surface, while the GNPs coated with Fc-binding secondary antibodies were used as detection reagent during the post-sample injection (Figure S3). The conjugate was synthesized by a one-step reaction in PBS, of which 500 µL functionalized GNP (10 nM) was added into 500 µL Stx1-1Ab solution (~670 nM). This mixture solution was sonicated for 1 min and vortexed for 1 hr. The solution was centrifuged under 8000 relative centrifugal force (RCF) for 5 min, the top supernatant was removed, and the pellet at the bottom was resuspended by adding 1 mL 1% PBS pH 7.4 and 1 µL Tween 20. Then this solution was washed by centrifugation method 3 times but using 1 × PBS to resuspend. It could be stored at 4 °C for two weeks for the SPRi injection.

### 4.4. Surface Plasmon Resonance Imaging

The antibody-modified biochip was mounted inside the SPRi optical chamber, using an 810-nm LED light source, and a charge-coupled device (CCD) camera as the signal detector, and the flow cell system controlled by a 6-way valve (Openplex, Horiba Scientific, Edison, NJ, USA). Each antibody spot shown in the real-time SPR image was labeled according to the microarray pattern. The DI water and PBS pH 7.4 buffer were injected at high flow rate of 500 µL/min to clean the tubing system and remove any BSA residue on the biochip surface. The reflectivity variations across the sensor surface were calibrated by injecting 200 µL of 20 mM PBS buffer at 50 µL/min.

At the beginning of kinetic measurements, the running buffer (PBS) was injected 3 times to stabilize the signal. The flow rate was set as a constant of 50 µL/min before and after the sample injection, and 3-minute pause-flow injections were used during sample injection to increase the reaction time of the antibody binding before the toxoid solution was flushed out of the flow cell. As described above, during the first one min of sample injection, the flow rate was at 50 µL/min, and then the pump was stopped for 3 min and resumed to 50 µL/min. For the GNP post-injection amplification, the GNP-antibody stock solution was diluted 10 times with PBS pH 7.4, the final GNP concentration was around 1 nM, and the number of Stx1-1 antibody molecules attached on each particle were estimated to vary from several to dozens. The conjugate injection flow rate and volume were set as the same values as the toxoid sample injection but without the pause, since the injected GNP showed much stronger ΔR.

The signals displayed during the experiments had two major parts, the real-time SPR images of the biochip surface including all of the printed spots and the background area, and the SPR sensorgram showing the real-time reflectivity changes of these spots along time. In the data analysis, the “difference images” shown in later sections were the automatically reconstructed images generated by subtracting the Stx antibody spots from control spots. Similarly, the SPRi sensorgrams shown in later sections and the signal values measured (ΔR) were from the automatic treatments using the sensorgram of antibody spots subtracted by the ones of the control spots. The real-time image treatment, ΔR subtraction, and sensorgram display were conducted by the SPRi View software (Horiba Scientific, Edison, NJ, USA).

After the SPRi detection was completed, two types of regeneration protocols were used for antibody screening/selection and GNP signal amplification. For the antibody screening and selection without using GNP, 100 mM NaOH was injected under the flow rate of 100 µL/min for 2 min. Later, for the post-injection amplification experiments with GNP, the regeneration took three steps as (1) 0.05%(*v*:*v*) Tween 20 in DI water, (2) 50 mM sodium dodecyl sulfate (SDS) + 20 mM NaOH in DI water at pH 12, and (3) 100 mM glycine + HCl in DI water at pH 3.0 were mixed with dimethylformamide (DMF) to the ratio 9:1(*v*:*v*). Each step took 10 min.

## Supplementary Materials

The following are available online at https://www.mdpi.com/2072-6651/12/5/280/s1, Figure S1: Functionalized GNP and GNP-antibody conjugate designed and synthesized to amplify the SPRi signal of Stx. Figure S2: Biochip fabrication protocol 1 and 2 for antibody spotting on biochip surface. (**A**) Protocol 1 used for direct label-free detection of Stx. (**B**) Protocol 2 used for sandwich immunoassay with GNP. Figure S3: Sandwich immunoassay designed for Stx detection with GNP signal amplification.
