# Supplementary material for: Rapid and Label-Free Immunosensing of Shiga Toxin Subtypes with Surface Plasmon Resonance Imaging
Source: Toxins (Basel). 2020 Apr 26;12(5):280. doi: 10.3390/toxins12050280 (PMC7291228; doi:10.3390/toxins12050280)
Supplement: Supplementary file 1 [file toxins-12-00280-s001.pdf]

Supplementary Materials: Rapid and Label-Free Immunosensing of Shiga Toxin Subtypes with Surface Plasmon Resonance Imaging

Bin Wang, Bosoon Park, Jing Chen and Xiaohua He

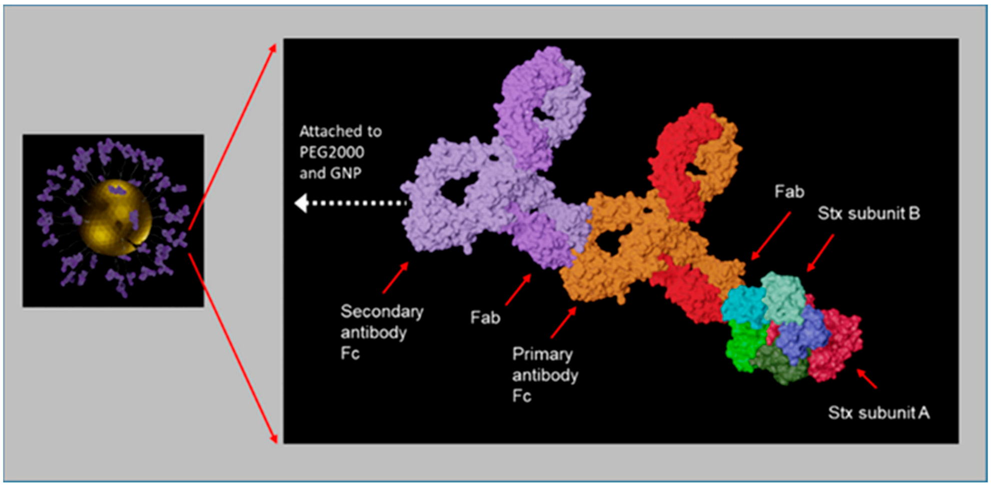

Figure S1. Functionalized GNP and GNP-antibody conjugate designed and synthesized to amplify the SPRi signal of Stx. In real experiments, Stx toxoids were used as less toxic alternatives of Stx proteins.

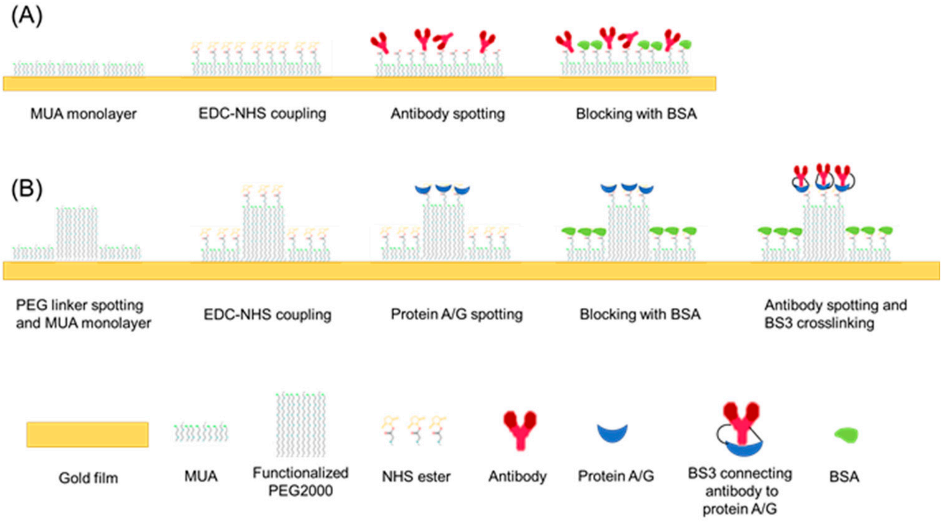

Figure S2. Biochip fabrication protocol 1 and 2 for antibody spotting on biochip surface. (**A**) Protocol 1 used for direct label-free detection of Stx. (**B**) Protocol 2 used for sandwich immunoassay with GNP. In real experiments, Stx toxoids were used as less toxic alternatives of Stx proteins.

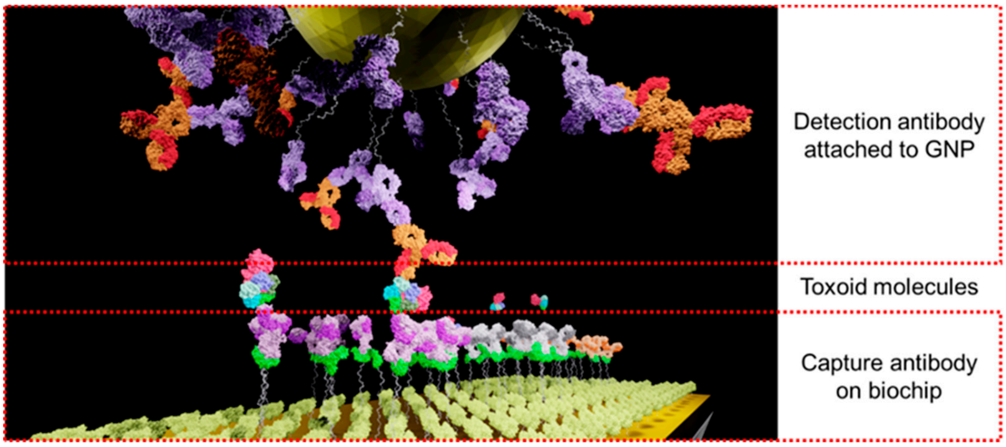

Figure S3. Sandwich immunoassay designed for Stx detection with GNP signal amplification. In real experiments, Stx toxoids were used as less toxic alternatives of Stx proteins.
